# Supplementary material for: A new species of parrot snake, Leptophis (Serpentes: Colubridae) from the Brazilian Cerrado
Source: PeerJ. 2025 Jan 30;13:e18528. doi: 10.7717/peerj.18528 (PMC11787803; doi:10.7717/peerj.18528)
Supplement: Supplemental Information 2 — Asterisk denotes specimens for which we also examined the hemipenes (*) or skulls (**). In some cases we only examined the skulls (***). We write the country and state names in capital letters and separate the locality names with commas and the state names with a semicolon. [file peerj-13-18528-s002.docx]

**Supplementary Material 1.** Specimens examined: Asterisk denotes specimens for which we also examined the hemipenes (*) or skulls (**) (in some cases we only examined the skulls***). We write the country and state names in capital letters and separate the locality names with commas and the state names with a semicolon.

***Leptophis ahaetulla* (n=492).** BRAZIL: AMAPÁ: no locality data: (CHUNB 3797, 22087, MPEG 19307), BR-156 highway, Água Branca stream: (MPEG 432), Campina Grande: (MPEG 16391), Macapá: (IBSP 24794, 24800, 24822, 45886), Macapá: IEPA: (MNRJ 8211), Oiapoque (IBSP 13778, 15385, 24852), Santana: Ilha de Santana: (IBSP 14806), Serra do Navio, Amaparí River: (MPEG 19684, MZUSP 4731). AMAZONAS: Barcelos: Parque Nacional do Jaú: Seringalzinho (INPA 9783), Borba (MNRJ 1552), São Gabriel da Cachoeira: Iauareté: Javari River: Estirão do Equador [a Brazilian military base]: (MPEG 148), Itacotiara: Javari River: (IBSP 44431, MCZ 2977), km 135 of the Manaus-Boa Vista road (=BR 174), km 218 of BR-174, 1 km E. of Reserva Indígena Waimari (MPEG 18992), Alexo Lake (=Aleixo, Manaus) (MCZ 2579), Manaus (CAS 49795, IBSP 18531, 32836, MNRJ 737), Manaus, Reserva Florestal Adolfo Ducke (INPA 10306, MZUSP 9833), Manicoré, surroundings of Passo Formoso farm (MPEG 20839), Maraã, Maguari (MPEG 18023, 18024), Presidente Figueiredo, Balbina Dam (IBSP 52002, 52198, MNRJ 8053, 9095, 9096, MZUFV 392, MZUSP 9648), Presidente Figueiredo, Balbina Dam, HPP flood area, Base 1, right bank of the Uatumã River (MPEG 17375**, 17376*, 17377–17381, 17394, 17416, 17493, 17497**, 17498, 18413), Reserva de Desenvolvimento Sustentável Amaña (INPA 9519), Vaupés River (IBSP 32010, MPEG 18), Tapurucuara (MZUSP 5470), Vila Nova (IBSP 14807). RORAIMA: Boa Vista, Coronel Mota Colony, Traiano region (MPEG 477, 494), BR 174, landmark 8 of the Brazil-Venezuela border (MZUSP 8555, 8556, 10302), Cujubim Waterfall, Catrimani River (MZUSP 8067), Confiança Colony (MZUSP 9985, 9986). MARANHÃO: Alcântara (HUFMA 281), Sapucaia Village, BR-226, about 60 km from Barra do Corda (MPEG 15015, 15563), Arari, Gancho do Arari (MPEG 13480, 14630, 15034, 15623, 15625, 16067 **, 16073, 16074, 16155, 16157), Carutapera (MPEG 18741), Igaranha Island (probably Ingarana, a village of Esperantinópolis) (IBSP 54987), Itapecuru-Mirim (IBSP 31955), Nova Vida, 25 km before the Gurupi River, between this and Maracaçumé Village (MPEG 11128–11130, 11163, 12279, 12281–12283, 13740–13742, 14789, 14790, 14793, 14795, 15288*), Peri-Mirim ( IBSP 21749, 21752), Puraqueú (a district of Igarapé do Meio), BR-222 (MPEG 15258, 16180, 16181), Santa Luzia do Paruá, BR-316 (MPEG 10837, 12031*, 12843, 13589, 13591–13593), Santa Maria, BR-226, between Grajaú and Barra do Corda (MPEG 15009, 15231, 15573, 16136), Santo Amaro do Maranhão (IBSP 75167, 75168), São José de Ribamar, Panaquatira Beach (HUFMA 169), São Luís, UFMA Campus (HUFMA 196), São Raimundo, BR-316 (MPEG 10879), Vitória do Mearim, Posto Nossa Senhora de Nazaré (MPEG 16093), Urbano Santos (HUFMA 282–291), Urbano Santos, Bom Fim Farm (HUFMA 51), Urbano Santos, Santo Amaro Farm (HUFMA 229, 234, MPEG 20541, 20542). PARÁ: Acará, Combú Island (MPEG 19306), Almeirim (LPHA 1220), Almeirim, Jari River (MPEG 16355), Ananindeua (MPEG 80, 16427, 16835), Ananindeua, Pirelli Farm, Uriboca (MPEG 18471), Augusto Corrêa, Cacoal (MPEG 572, 3202, 3209, 3804, 3806, 3808, 5407, 5409, 5418, 5429, 6505, 6514, 6515, 6463, 6671, 6683), Aveiro (LPHA 1259), Barcarena (KU 128105, 129879, MPEG 15453, 16567), Belém (AMNH 56157, BMNH 1970.506, IBSP 17674, 23815, 25435, 25442, 53896, MPEG 18375, 18546, 18624, UFRGS 1053), Belém, Bosque Rodrigues Alves (MPEG 18626), Belém, Campus da UFPA (MPEG 450, 621), Belém, Campus da UFRA (MPEG 19839), Belém, Campus do MPEG (MPEG 18457, 19836, 20507, 21183 **), Belém, Mosqueiro Island (MPEG 1113, 1526, 2598, 13277, 17690, 18630 *), Belém, Outeiro Island (MPEG 21043), Belém, Utinga (KU 124601, 124603), Santa Bárbara, Genipaúba (MPEG 2629, 3285, 6033, 6952, 8430, 18882), Bragança, Bom Jesus (MPEG 5048, 5060, 6202, 6214, 6241, 7926, 7933, 7950, 8346, 11378 ), Cametá (IBSP 2225), Canindé, Gurupi River (MZUSP 4255), Capitão Poço, Boca Nova (MPEG 1626), Capitão Poço, Santa Luzia (MPEG 1612, 2904, 12175, 12179, 12181), Castanhal, Boa Vista ( MPEG 513, 594 ***, 1164, 1165, 1473, 1483, 1554, 2712, 4014–4016, 5719, 5721, 6928), Castanhal, Macapazinho (MPEG 5861, 9461, 10773, 11796), Conceição do Araguaia (IBSP 23577), Curuçá, Marauá (MPEG 7617, 5887, 5899, 7128, 7129), Igarapé-Açú (MPEG 893, 937), Itaituba (LPHA 596, 863, 1426, 1427, MCP 4416, 4433), Jacaréacanga (MNRJ 9054, 9055), Ilha de Marajó (IBSP 17649), Ilha de Marajó, Breves-Anajás, km 8 of the PA-158 (MPEG 17348, 17351), Ilha de Marajó, Cachoeira do Arari (MPEG 18016, 18326), Ilha de Marajó, Chaves, Marajá Farm, (MPEG 17722 *), Ilha de Marajó, Chaves, Tijuquara Farm, Tijuquara River (MPEG 17714), Ilha de Marajó, Muaná (MPEG 19330, 19332–19334), Ilha de Marajó, Salvaterra (MPEG 18637, 18638), Ilha de Marajó, Santa Cruz do Arari (MPEG 19627, 19628), Jacundá to Itupiranga, BR-230, Transamazônica (IBSP 40019), km 16 of the PA-252(MPEG 8075, 13286, 15192), Marabá, km 11 of PA-222, formerly PA-70 (=BR-222 ) (MPEG 7462, 7467, 9489, 15164), Parauapebas, Serra Norte (KU 124602), Marituba, Parque Verde (MPEG 18549), Melgaço, FLONA de Caxiuanã, laranjal community, Laranjal stream (MPEG 19739, 19740), Melgaço, FLONA de Caxiuanã, Estação Científica Ferreira Pena (MPEG 19735, 20122), Óbidos (MZUSP 1255), Oriximiná (MPEG 3268, MZUSP 5485), Oriximiná, Boca do Cuminã-Mirí (MZUSP 4820–4822), Oriximiná, Porto Trombetas (MNRJ 16805, MPEG 19774), Ourém, Limão Grande (MPEG 1637, 1639, 2166, 6173), Palestina do Pará, Porto Jarbas Passarinho, Araguaia River (MPEG 11765, 12751, 12957, 12958), Parauapebas, Carajás, Serra Norte, N5 area (MPEG 16927), Parauapebas, bank of the Parauapebas River (MPEG 16599), Peixe-Boi (MPEG 684, 1415), Prainha (LPHA 2579), Tapajós River (MCZ 3288), Santarém (LPHA 80, 350, 362, 405), Santarém, Cucuruna, Ramal do Coelho (MCP 7575), Santarém, road to Cachoeira do Palhão (MPEG 303), Santarém, Tapari, right bank of the Tapajós River (LPHA 1778), Serra do Cachimbo (IBSP 18529, MZUSP 3347), Fresco River, right bank of the Gorotide River (MPEG 17021), Santa Bárbara, Morelândia Farm (MPEG 18480), Santarém Novo, Trombetinha (MPEG 3478, 4155, 4782), Santo Antônio do Tauá (MPEG 1874, 5458, 5700, 6954, 19434 *, **), Tomé-Açú (IBSP 14839, 14842), Tucuruí (IBSP 46887, 46905, 47088, 47324), Tucuruí, right bank of the Tocantins River (MPEG 16722), Viseu, Colônia Nova, near the Gurupi River, BR-316 (MPEG 4330, 5161, 10303, 12922, 13895, 13896, 13926–13928, 13931, 15852, 15854), Viseu, Curupati (MPEG 10042, 10899, 14447, 16007, 16008), Viseu, km 220 of BR-316 (MPEG 1670, 2207, 9575), Vitória do Xingu, Belo Monte Dam, Arroz crú, left bank of the Xingu River (MPEG 19872). PIAUÍ: Gilbués (MPEG 21695), Piripiri (MNRJ 11364), Simplício Mendes (IBSP 42453), Sítio Ouro Verde, PARNA de Sete Cidades (MPEG 21697), Teresina (IBSP 503, MPEG 21696), Valença do Piauí (MZUSP 5816). RONDÔNIA: Alto Alegre dos Parecis, Line P-30, km 13, Santa Maria Farm (UFMT 7019), Espigão d’Oeste (IBSP 71634, MHNCI 9592), Ji-Paraná, km 11 of line 12-B (right side) (MPEG 16869), Nova Brasília (MZUSP 8516), Nova Colina, Polonoroeste (MZUSP 8506, 8507), Nova Esperança (MZUSP 8363, 8520), Ouro Preto do Oeste (CZGB 2875, MPEG 16830), Porto Velho, Samuel Dam, BR-364 (MPEG 17755*,** MPEG 17758). MATO GROSSO: Upper Araguaia River (CHUNB 65868), Apiacás (MZUSP 11175), Barra do Tapirapé (AMNH 93593–93595), Brasnorte, PHC Bocaiúva (UFMT 8058), Campo Novo do Parecis, PCH Baruíto, Sangue River (UFMT 4568), Lambari D´Oeste (MNRJ 24929), Lucas do Rio Verde, Córrego stream (UFMT 8769), Paranaíta, M2-P3, Module 2, Castanheiro (UFMT 8523), Vale de São Domingos, Guaporé Dam (UFMT 2142*, 2143), Vila Bela de Santíssima Trindade (MZUSP 11433). TOCANTINS: Lajeado (IBSP 64266, 64270, 64351, 64396, 64514, 64537, 64538), Miracema do Norte (=Miracema do Tocantins) (IBSP 66422, 64423*,**), Palmas (CHUNB 23620, IBSP 65623, 65907, 66317), Palmeirante, Porto da Balsa Palmeirante-Goiatins (UFMT 8605), Peixe (CHUNB 52568–52573, 52598), Luís Eduardo Magalhães Dam (MZUSP 14179, 14181, 14182). GOIÁS: Araguatins (MZUSP 3835), Aruanã (MZUSP 2174), Pium (CHUNB 24750), Santa Isabel, Bananal Island, Araguaia River (AMNH 90336), São Salvador do Tocantins (MZUSP 12127). GUYANA: DEMERARA-MAHAICA: Berbice, Cow Falls on Corentyne River (CM 44305), Essequibo (FMNH 26662). GUYANA: Dunoon, Demerara River (UMMZ 47762, 47763, 149833***), Dubulay ranch on the Berbice River (AMNH 140899), Georgetown (UMMZ 80502), Essequibo, Pomeroon (USNM 84518, 85087), Georgetown, East Demerara, 50 mi above, on the Abary River (USNM 141762), vicinity of Georgetown (AMNH 9624). CUYUNI-MAZARUNI: Kartabo (AMNH 15152, 15153, 18176–18178, 44906, 98200–98202, FMNH 14839), MAZARUNI-POTARO, near Kartabu (AMNH 21281, 137331), Mazaruni-Potaro, WSW of Mabura Hill, ca. 25mi, near Magdalen’s creek camp, NW bank of the Konawaruk River (USNM 566268), Rockstone, Essequibo River (FMNH 26661), Rupununi Savannah (BMNH 1933.6.19.55), Wismar (AMNH 61559). BARIMA-WAINI: Aremu River (UMMZ 56427), Kumaka (USNM 164218), 5 mi of Baramita, Golden city mining camp (USNM 535809, 535810). BARIMA-WAINI REGION: Rupununi, N of Akarai Mountains, W of New River (KU 69831), on road of Mabaruna (UTA 50170). FRENCH GUYANA: CAYENNE: (MCZ 1980, MNHN 1989-3077, USNM 451). SAINT-LAURENT-DU-MARONI: no locality data: (MNHN 1895-18). SURINAME: no locality data: (IBSP 13762, USNM 6116). BROKOPONDO: Brownsweg-Paranan Rd, 3 Km out of Brownsweg (MCZ 152598), Sara Creek (AMNH 104616). COMMEWIJNE: Nieuw Amsterdam (MZUSP 4541, 11292). MAROWIJNE: Langamankondre (MZUSP 4543), Tepoe (UTA 15862–15864*–15867), Moengo (USNM 64625), Paloemeu (USNM 159052, 159053). CORONIE: Paramaribo-Nickerie Rd, 15 km W of Baskamp (MCZ 154840), 170 km W Paramaribo, W Coppename River, Paibo-Nickerice Rd (MCZ 152596). NICKERIE: Paramaribo-Nickerie Rd, 7 km W Henar (MCZ 156343). PARA DISTRICT: Lelydorp (located in the WANICA DISTRICT), De Craneweg nº 16 (KU 221553, 221554). WANICA DISTRICT: Kwatta (CM 44304). PARAMARIBO: Paramaribo (AMNH 4425, 8131, 8147, 8687, 8688, 97715, 104614, 104615, BMNH 1946.4.4.16, BMNH 1946.4.4.17, BMNH 1946.4.4.18, KU 206417). SARAMACCA: La poole (La Poule) (FMNH 134734), Raleigh Cataracts, Coppename River (AMNH 108799). SIPALIWINI: Raleighvallen-Voltzberg Nature Reserve, Foenjoe Island, Coppename River (MCZ 152204), somewhere up the Coppename River (AMNH 73843), 86 km from Moengo on Moengo-Paramaribo stretch of Albina-Paramaribo Rd (MCZ 152597). VENEZUELA: AMAZONAS: Caño San Miguel, 50 km from the mouth of Guainia River (MHNLS 11606), San Antonio, upper Orinoco River (USNM 83617), Siapa River (MHNLS 14011**). BOLÍVAR: Aza (MHNLS 14656). COLOMBIA-BRAZIL boundary: Carurú, upper Vaupés River (AMNH 4463, AMNH 4464). VENEZUELA-BRAZIL boundary: Amazonas, Salto do Huá, Maturacá River (USNM 83564, USNM 83570), Santa Isabel (AMNH 38097).

***Leptophis bocourti* (n=16).** ECUADOR: ESMERALDAS: Durango (QCAZ 8022, QCAZ 8023), Molinitos Farm, vía La Tola (QCAZ 3856), La Mayronga, Lagarto (QCAZ 2236), Misión Santa María de los Cayapas (QCAZ 320), Reserva Ecológica Mache-Chindul, Laguna de Cube (QCAZ 16942), Saint Javier (San Javier), W Ecuador (BMNH 1901.3.29.30), San Mateo (FMNH 28054, 28056), Zapallo Grande (MZUSP 8250). IMBABURA: La Carolina San Miguel de Ibarra (UMMZ 83709–83711, 149838***), Paramba (a farm) (BMNH 1946.1.6.67). PICHINCHA: 47 km S of Santo Domingo de los Colorados, Centro Científico Palenque River (USNM 285488).

***Leptophis bolivianus* (n=38).** BOLIVIA: BENI: Estancia Yutiole, ca. 20 km S San Joaquim (AMNH 104562–104564*,**), Puerto Capitán Vasquez, Itenez River (AMNH 101883), Rurrenabaque (AMNH 22446), Santa Rosa, Mamoré River (AMNH 101881, AMNH 101882), Vaca Díez, Tumi Chucua (USNM 280392–280394), upper Beni River (ANSP 11335). SANTA CRUZ: no locality data: (IBSP 33287, MNHN 7306), Buena Vista (BMNH 1927.8.1.183, CM 2702, FMNH 35614–35621, MCZ 27553, UMMZ 60701, 60702, 60709, 67973–67976a, 67976b, 67977a, 67977b, 149834***), Sara, Las Yuntas (CM 23), San Antonio de Parapetí (AMNH 141443). TARIJA: Bermejo (FML 591).

***Leptophis coeruleodorsus* (n=79)*.*** GUYANA: no locality (AMNH 8718, 9022–9024). TRINIDAD: no locality data: (AMNH 73146***, USNM 5587, 15235, 17746, 59931–59933, 60598), Arima (USNM 166715), Arima, Arima Valley (AMNH 77030, 119443**), Arima-Blanchisseuse Road, ca. 2 mi S Simla-Quarry Road (FMNH 215817), Arima-Blanchisseuse Road, 21 miles marker (FMNH 219581), Arena (AMNH 73094), Blanchisseuse, 5 mile post on Blanchisseuse Road (AMNH 81477), Caroni Swamp (MCZ 100656), Cumuto (AMNH 81479), Diego Martin (MCZ 126381), Guayaguayare (AMNH 81474, 81476), Mayaro, Plaisance (FMNH 49936), Santa Cruz Valley (MCZ 100651*, 100652), N Arima, Morne Bleu (FMNH 49937), Port of Spain (AMNH 101309, BMNH 1964.1979), Port of Spain, Cascade Road, Saint Anns (AMNH 81475), Port of Spain, Golf Course Road (AMNH 85946), Sangre Grande, Rio Grande Forest, below Tree Station (AMNH 81478), Tucker Valley (AMNH 64478a, 64478b), Vega de Oropouche, Esperanza Estate (AMNH 85945), 3.1 mi N junction Toco Road and Valencia Road, on Toco Road (FMNH 217234), between Los Bajos and Palo Seco, Siparia-Erin Road 22-25 mile mark (FMNH 218776). TOBAGO: no locality data: (UMMZ 90671), ca. 0.75 NNW of Speyside, on windward Road, Milepost 25.1 (USNM 228065), Charlotteville (USNM 195163), near Milford Bay, SW Tobago (MCZ 11994, 11995, 12026), Saint Augustine (BMNH 1964.1991), Saint John, Charlotteville (USNM 228064), Ward of Tobago, Cambleton (USNM 195127*), Ward of Tobago, near Pembroke (AMNH 110472), Saint George, Mount Saint Benedict, Tunapuna (CM 6491, 6540). VENEZUELA: no locality data: (ANSP 5182). Caracas: El Valle (AMNH 59402, 59403), Caguital, El Limon Farm (MHNLS 4694). AMAZONAS: Cerro de La Neblina, ca. 6.2 km NNE of Pico Phelps, Camp XI (USNM 562695), Rio Negro, vicinity of Neblina, base Camp on left blank of Baria River (= Nawarinuma River) (USNM 562694), 56 km NNW of Esmeralda, Cunucunuma River, Belen (USNM 217213). APURE: 6 km W San Fernando de Apure (TCWC 46261, 46262*, 46263). BOLIVAR: El Manteco (AMNH 114754), Hato La Yeguera, 32 km E E1 Manteco (AMNH 114753), 16 km S. El Dorado, in a goldmine (AMNH 138468), 22 km E El Palmar on road to Rio Grande (MVZ 176257), 61 km N El Manteco (AMNH 114752). CARABOBO: Bahia de Patanemo, Puerto Cabello (MHNLS 6286). COJEDES: Hato Itabana, 38 km, 5.0 de Las Vegas, 80 m (MHNLS 6446). GUARICO: Caraquito (ANSP 18288), 15.6 km N Altagracia de Orituco, HWY 12 (TCWC 58709). MIRANDA: Chico River, 100 mi E of la Guaira (USNM 27831), Santa Lucía, 11 km ENE on Siquire stream (CM 7433). MONAGAS: Aragua de Maturín, Limon, near Rancho Grande (AMNH 98446), Caripito (AMNH 98263, 98264, TCWC 59756), Sotillo, near Uracoa, Maturin Savanna (CM 17387), Laguna, Guanipa, Reserva Forestal Guarapiche (MHNLS 13595). SUCRE: Los Arroyos, Benitez (MHNLS 13334). DELTA AMACURO: Cano Macareo, Delta Medio (MHNLS 13306).

***Leptophis dibernardoi* (n=41).** BRAZIL: CEARÁ: Aiuaba (URCA 5539), Aquiraz (CHUFC 1602, CHUFC 1227, MCP 18318, Barro (URCA 6127, URCA 6142, URCA 7890), Crato (URCA 10126), Farias Brito (URCA 9431, URCA 10617, URCA 10618), Fortaleza, Campus do Pici (CHUFC 1140, CHUFC 1722, CHUFC 1980), Messejana, Fortaleza (CHUFC 1104), Mucuripe (MNRJ 1959–61*), Guaiuba (MPEG 27110, ZUFMS-REP 3456, CHUFC 1929*), Iguatu, Chapada do Apodi (CHUFC 525*, MCP 17835*), Lavras da Mangabeira (URCA 11130), Limoeiro do Norte (CHUFC 221, 365, 493, 561), Maranguape, Serra de Maranguape (MZUSP 23131, CHUFC 1172, CHUFC 1244), Mauriti (URCA 12023), Morada Nova (CHUFC 1721), Pacajus (CHUFC 1732), Trairí (URCA 5696). PIAUÍ: São Raimundo Nonato, Parque Nacional da Serra da Capivara (MNRJ 7596). RIO GRANDE DO NORTE, João Câmara (URCA 1245). PARAÍBA, João Pessoa, Jardim Botânico Benjamim Maranhão (UFPB 4300*). PERNAMBUCO, Exu (URCA 6436). MINAS GERAIS, Jaíba, Mocambinho (MZUFV 842, MZUFV 913).

***Leptophis liocercus* (n=72).** BRAZIL: no locality data (AMNH 3531). ALAGOAS: Murici, Serra Branca Farm (MNRJ 3969), São Miguel dos Campos (MNRJ 3955, 3974). BAHIA: Almadina, Bom Fim Farm (MZUESC 120), Barro Preto (= Governador Lomonto Jr.) (CZGB 3301), Belmonte (CZGB 4434, 7530), Camamu (MZUESC 326, 1082, 3032, CZGB 8643), Canavieiras (CZGB 2062), Caravelas (IBSP 966), Coribe (CHUNB 6640*), Gandú (CZGB 3878), Governador Lomonto Junior (CZGB 3255, 3406, 3437, 4457), Ibicaraí (CZGB 8987, MZUESC 241**, 291, 741), Ilhéus (CZGB 108, 281, 555, 1149*,** 3160, 3243, 3436, IBSP 54201, MZUESC 2002*), Itabuna (CZGB 380, MZUSP 1273), Itagibá (CZGB 6795), Itaparica (IBSP 52353), Itapebi (CZGB 6960, 8765), Itiúba, Bela Conquista Farm (CZGB 8715), Jaguaquara (CZGB 9255), Jaguarari (IBSP 33352), Juazeiro to Curuça (MZUSP 10629), Mucuri, Conjunto Boa Sorte (CZGB 3984), Mutuípe (CZGB 6562, 8200), Poções (IBSP 67645), Porto Seguro (IBSP 54273), Santa Clara, Mucuri River (MCZ 2989). ESPÍRITO SANTO: Train Station of Baixo Gandú (IBSP 8271), Domingo Martins, Estação Araguaia (IBSP 8403**), Porto Cachoeiro de Santa Leopoldina (MZUSP 734), Sítio Goaporini (MNRJ 4846*,**), Vitória (MCZ 2978). MINAS GERAIS: Pedra Corrida Train Station (IBSP 9477, 9612**), Várzea da Palma (MNRJ 6672). PARAÍBA: Campina Grande (IBSP 51324), João Pessoa (UFRGS 1051), Mamanguape (CHUNB 29019). PERNANBUCO: Recife (IBSP 17301), Recife, Dois Irmãos (MCN 5340*, 5671), Moreno, Tapera (MCN 5648). RIO DE JANEIRO: Cabo Frio (IBSP 21089), Duque de Caxias (MZUSP 1940), Macaé (MZUSP 4214), Rio de Janeiro (USNM 120830), Rio de Janeiro, Parque Natural Municipal da Serra do Mendanha (MNRJ 10966, 10967), Rio de Janeiro, Pedra de Guaratiba (MBML 285). SERGIPE: Aracajú (IBSP 46053**).

***Leptophis marginatus* (n=178).** ARGENTINA: FORMOSA: Formosa (FML 2034), Laishi, Misión Laishi, Reserva Ecológica El Bagual (FML 11273, 11274, 11292, 11304, 11568, 11569, 11570, 11571, 11572, 15924), Laishi, Puerto Cano (MHNCI 4795), Pilagás (FML 1628), Pilcomayo, Puerto Pilcomayo (FML 1624*,**). JUJUY: Ledesma, Yuto (FML 598, 619), Zapenaga River, border of the Chaco de Santa Fé and the grand Chaco of Jeaño (MNHN 1904-98). SALTA: General José San Martin: Acambuco strem, 7 km W Itiyuro Dam (FML 1076), Piquirenda (FML 461). BOLIVIA: SANTA CRUZ: Puerto Suárez (CM 314). BRAZIL: MATO GROSSO: Araputanga, Ombreiras, left bank, setor 2 (UFMT 2814), Aripuanã, Bairro Ribeirão do Lipa (MZUSP 11143, UFMT 1721, UFMT 4250), Barão de Melgaço, Baia das Pombas Farm (ZUEC 568), Barão de Melgaço, São Francisco do Perigara Farm (UFMT 252), Barra do Tapirapé (AMNH 93593, 93594, 93595), Cáceres (IBSP 49061), Cáceres, Cotriguaçú, São Nicolau Farm (UFMT 8998), Chapada dos Guimarães (CHUNB 15440, 15441*,**15452**–15457, 20423*,**20425, UFMT 2306), Chapada dos Guimarães, Manso (UFMT 628, 629), Cuiabá, Bairro Boa Esperança (UFMT 168, 2153), Cuiabá, found at km 8 of the Santo Antônio de Leverger road (UFMT 2349), Cuiabá, km 5 of Rodovia Palmiro Paes de Barros (UFMT 8633), Cuiabá, Flor do Cerrado allotment, km 8 of Rodovia Emanuel Pinheiro (UFMT 8780), Cuiabá, bank of the Cuiabá River, about 5 km from Guia (UFMT 79*), Cuiabá, Santa Amália (UFMT 8536), Poconé-Porto Cercado road (MZUSP 7310), Cuiabá, Ribeirão do Lipa, São Sebastião Farm (UFMT 5473, 5506), Jauru, PCH Ombreiras, right bank, sector 3 (UFMT 2816), Nossa Senhora do Livramento, Babaçual, creek along the MT-60 Highway (UFMT 2145), Nossa Senhora do Livramento, Baía do Coqueiro, Pirizal (UFMT 2221), Nossa Senhora do Livramento, Pirizal (UFMT 3541, 8557), Poconé (MHNCI 7910), Poconé, MT-60 (UFMT 6763), Poconé, Boa Vista Farm (UFMT 40), Poconé, Santa Inês Farm (ZUEC 1233), Poconé, Garimpo do Alemão (UFMT 2202), Poconé, Rodovia Transpantaneira, km 11 (UFMT 255), Porto Esperidião (MZUSP 6413), Porto Estrela, Headquarters of the Estação Ecológica Serra das Araras (UFMT 8321), Porto Velho, Rio Tapirapés (MZUSP 3764), Santo Antônio de Leverger (UFMT 5809), Serra do Roncador (BMNH 1972.414), Manso Dam (MZUSP 11939, 11949), Vale de São Domingos, Guaporé Dam (UFMT 2144, 2146). MATO GROSSO DO SUL: Anastácio (IBSP 50247), Anaurilândia (IBSP 59556*, 59557), Aquidauana (IBSP 18470), Campo Grande (IBSP 19628, 26764), Corumbá, Base de Estudos do Pantanal (ZUFMS-REP 1171), Corumbá, BR-262 (ZUFMS-REP 2208), Corumbá, Serra do Amolar, Gaíva Farm (UFMT 1437, 1491), Corumbá, Serra do Amolar, Lagoa Mandioré (MNRJ 216, 339), Corumbá, Serra do Amolar, RPPN Acurizal (UFMT 1428, 1433, 1435), Corumbá, Serra do Amolar, RPPN Penha, Moquém stream (UFMT 1424), Dourados (IBSP 27587, 44058), Dourados, Itahum (IBSP 16722), Itaquiraí (IBSP 49908), Miranda (USNM 100748), Miranda, Agachi (IBSP 14278, 14601), Miranda, Salobra (UMMZ 109052*), Miranda, Retiro Piúva (na airport) (MZUSP 8256, 8259), Ponta Porã (IBSP 16427, 31831, 41146, 42244), Nioaque (ZUFMS-REP 1397), Correntes River (IBSP 7620). ESPÍRITO SANTO: Aracruz, Santa Cruz (MBML 130), Itapina train station, Colatina (IBSP 8989, 9121*). PARANÁ: Altônia (MHNCI 7440), Boa Vista da Aparecida (MHNCI 8548**), Capitão Leônidas Marques, Andrada River (MHNCI 8423, 8424), Diamante do Norte (MHNCI 8014, 8017, 8221, 11717), Diamante do Norte, Estação Ecológica Caiuá (MHNCI 10559), Diamante d’Oeste (MCP 16239), Foz do Iguaçú (IBSP 44678, 44688, MHNCI 3871, 11781), Guaíra (IBSP 32851, MHNCI 632, 633, 2773, 2855), Itambé (MHNCI 8109), Ivaí River (MCN 3371), Nova Prata do Iguaçú (MHNCI 2246), Ouro Verde do Oeste (MHNCI 10356), Palotina (MHNCI 8100), Pato Bragado (MHNCI 10355), Toledo, PCH São Francisco (ZUFMS-REP 989), Três Barras do Paraná (MHNCI 8422), Ivaí River Valley (MHNCI 8303, 8304, 8305). RIO DE JANEIRO: Cascadura train station (IBSP 9019), Rio de Janeiro (MZUSP 2339), Manguinhos (UMMZ 109053). SÃO PAULO: no locality data: (UMMZ 62803, 149835***), Ilha Solteira (IBSP 36961, 38192, 38966), Pereira Barreto (IBSP 54055**, MHNCI 4512**), Presidente Epitácio, Rio do Peixe (IBSP 63731), Santo Anastácio (IBSP 37444*), Teodoro Sampaio (ZUEC 966, 995*), Engenheiro Sérgio Motta Dam (IBSP 60028). PARAGUAY: no locality data: (ANSP 5514). ASUNCIÓN: (IBSP 10092). AMAMBAY: Parque Nacional Cerro Cora, near Comandancia (CM 94311). CANINDEYÚ: Colonia Indígena Chupa Pou, about 35 km N Curuguaty (AMNH 143299), Lagunita, Reserva Natural del Bosque Mbaracayú (TCWC 90574). CORDILLERA: 12 km N Tobatí by road (MVZ 110989*). ITAPUÁ: Capitán Miranda, Hotel Tirol, 18 km E Encarnación, Route 6 (CM 109293), vicinity of El Tirol, 19.5 km N of Encarnación, on highway 6 (USNM 253553). SAN PEDRO: no locality data: (FML 637). PRESIDENTE HAYES: Primavera, Alto Paraguay (BMNH 1971.430). URUGUAY: ARTIGAS: Arrocera Conti, 20 km W of Colônia Palma (ZVC-R 4401, 4529, 4530).

***Leptophis nigromarginatus* (n=373).** BRAZIL: ACRE: Brasiléia (MZUFV 71), about 5 km from Porto Walter (MZUSP 7355, 7370, 7371), Feijó (UFRGS 1052), Porto Walter, Juruá River (MPEG 20383**), Rio Branco (IBSP 54956), Rodrigues Alves, Rio Crôa (MZUSP 14116), Tarauacá (IBSP 18528, 18530**), 5 km N Rio Branco (MPEG 18249). RONDÔNIA: Alto Paraíso (MZUSP 8725), Porto Velho (AMNH 22254, CHUNB 33820, 66504–66507), Porto Velho, Abuña (UMMZ 56898), Guaporé River (IBSP 22156). AMAPÁ: Oiapoque, Oiapoque River (AMNH 58205). AMAZONAS: Alto Solimões (MCN 4163), “Amazon, Prauquiza, Cremarea” (CM 2007, 2008), Amazonas River (MCZ 156332), Benjamin Constant (MNRJ 738, 739), Boca do Acre (MZUSP 5751), Tefé River mouth, tributary of Solimões River (MNHN 1900-462, MNHN 1900-463), Igarapé Belém, near Solimões River, ca. 70 km E Letícia (AMNH 115022), Madeireira Scheffer, Ituxi River (MPEG 20333**), Manacapurú (MZUSP 8647), Manacapurú, Miriti River (AMNH 67956), Manaus (BMNH 97.12.29.15), Manjuru River (AMNH 114262), São Paulo de Olivença, Amazonas River (AMNH 56160), Tefé (MCZ 2958, 2980, IBSP 15060, IBSP 15080). PARÁ: Alenquer, Atuma (MCP 7636), Mojú Island, Japurá River (MZUSP 6602, 6603), Monte Alegre, Bom Jesus Farm, Curral Grande (IBSP 40514, 40830), Óbidos (MCZ 2584, UMMZ 56304a, 56304b), Prainha (LPHA 2566), Santarém (LPHA 1731*, MCZ 2792**, 2938), Santarém, Costa do Tapará (MCP 10605), Santarém, São Miguel Island(MCP 7905), Taperinha Farm (MZUSP 5187, USNM 120828, 120829). COLOMBIA: AMAZONAS: La Pedrera (IBSP 9186), Leticia (AMNH 126464, 126465, CM 55621–55623**, FMNH 83039, MCZ 48985, 145077, near Leticia USNM 307003), Santa Sofia Island, 20 mi NW Leticia (MVZ 164266), Santa Sofia Island, 35 km above Leticia (MCZ 141089), 6 km NW of Leticia (MVZ 172125), Apaporis River (MCZ 53229–53231), Gino-Goje, lower Apaporis River (MCZ 53232, 53233). CAQUETÁ: Morelia (ANSP 25646, 25647). MAGDALENA: Bonda (MCZ 11859). PUTUMAYO: San Antonio, Guamúez River (KU 140410). BOLIVIA: BENI: Beni River (AMNH 22267, 22268), vicinity of Riberalta (AMNH 22258, 22259), Vaca Díez, Villa Bella (CM 366). ECUADOR: NAPO: Aguarico River, SW mouth of Cuyabeno River (FMNH 218506), on road halfway between Puerto Napo and Misahualli (UMMZ 177889), 6 km E Tena, Fischer Farm, along Napo River (TCWC 65017, 67309), Johanna Farm, 3 km N Tena (TCWC 68730). PASTAZA: upper Bobonaza River (USNM 211030, 211031, 210032, 210033), Arajuno (UTA 37975). SUCUMBIOS: Cuyabeno, Napo River (MZUSP 9535), Limoncocha (KU 98629), Santa Cecilia (KU 105409, 107041, 107042, 112279, 126041, 126042, 121900–121902, 148344–148349, 148351, 155512, 155513, 175424, 183519). GUYANA: Karanambo (AMNH 60792). PERU: no locality data (ANSP 11350, 11351), upper Tapiche River, tributary of the Ucayali River (AMNH 56035). AMAZONAS: Cenepa River, Aguaruna Village (MVZ 163293). CAJAMARCA: 1 km N to NNW Monte Seco, Zaña River (FMNH 231776*). HUÁNUCO: Tournavista (AMNH 104294). JUNÍN: mouth of Tambo River, upper Ucayali River (AMNH 52900), Huachi Yaku, middle Morona (AMNH 52472), Norman Farm, Ipoki River, ca. 3mi Perene River (USNM 193809). LORETO: upper Curanja River, Champuia stream (MZUSP 3346), Balta, Curanja River (LSUMZ 26801, 26802, 26803, 26804, 26805), “Iquitos?” (AMNH 55908), Centro Union (TCWC 42177*), Calleria Colony, Calleria River, 15 km from Ucayali River (CAS 93208), Contamana (AMNH 53564), Contamana, Suhuayo Lake, Rean Rean (AMNH 52885) Contamana, Suhuayo Lake (AMNH 53010), Contamana, Suhuaya Island (AMNH 52296, 53008), Contamana, Santa Rosa de Chia Tipishca (AMNH 52165), Gamitana Cocha, lowerAmazonas River (FMNH 45570), Iquitos (AMNH 52044, 52045, 52049, 52121, 52137, 52153, 52230, 52320, 52419, 52421, 52501, 52508, 52518, 52520, 52529, 52530, 52597, 52598, 52629, 52647, 52653, 52669, 52723, 52732, 52733, 53047, 53074, 53154, 53226, 53271, 53391, 53396, 53397, 53402, 53411, 53412, 53610, 53648, 53685, 53711, 53759, 53829, 54084, 54134, 54153, 54210, 54216, 54225, 54233, 54335, 54345, 54644, 54715, 54810, 54894, 54940, 54976, 54980, 54984, 56037, 56106, TCWC 38195, 40539, 42175, 44657), Iquitos, Lupuna Island (AMNH 53225, 56092, 56093), Iquitos, Itaya River (AMNH 53684, 54116, 54865, 55076, 55080, 55125, 55140, 55211, 55246, 55273, 55278, 55311), Miranio Lake (AMNH 53299), Lower Pisqui River (AMNH 56026), lower Tigre River (AMNH 53185), Mishana, Nanay River (TCWC 38196, 39109, 39110, 42174, 44658, 44090, 44659), Montaña of Perea (FMNH 40031), Moropón (TCWC 38047, 38193, 38194, 39108, 42170, 42171–42173, 44660, 44661), mouth of Cushabatay River (AMNH 55954), Nauta Caño, Marañón River (KU 220505), near Iquitos, Quisto Cocha (LSUMZ 48419), Orellana (AMNH 52094, 52903, 54575, 54580, 54588, 54614, 55672), Pampa Hermosa, Cushabatay River (AMNH 53234, 53235, 53380–53382, 53430, 53504, 53513, 53518, 55384, 55349, 55430, 55431, 55436, 55454, 55460, 55461, 55479, 55480, 55727, 55728, 55734, 55742, 55745, 55751, 55755, 55762, 55799, 55803, 55962, 55964, 56007, 56013, 56015), Pampa Hermosa, Ucayali River (AMNH 52028), Paña stream, Cushabatay River (AMNH 52345), Paraiso (TCWC 42176), Pévas (ANSP 5200, 11634, CAS 12498), Pebas Pévas, Ampiyacu River, E Peru (CAS 8728), Peru-Brazil border on Tapiche River (AMNH 52192), Pévas (MNRJ 2982, 3018), Porolongo River, Sapa River (AMNH 52710), Samiria River and Parinari Canyon (AMNH 57287, 57288, 57289**, 57290, 57291, 57292, 57293, 57294, 57295), Tamaya River (AMNH 52334), Tamaya River, Ucayali River, Sobral (AMNH 55326), Tapiche River, Punga (AMNH 52080, 52082, 52084), Ucayali River (AMNH 53206, FMNH 45574, 56156, 56157, 56159, IBSP 14803, MCZ 3789, 3796), Sarayacu, Ucayali, NE Peru (BMNH 81.5.13.58), Shiriara, Nanay River (AMNH 56079), upper Amazonas River (BMNH 1946.1.5.7, FMNH 11184), upper Ucayali River (AMNH 71132, 71133), Yarinacocha (LSUMZ 26799, 26800*), 5 mi below Las Piedras River mouth (= Tacuatimanu River) (FMNH 83304). MADRE DE DÍOS: no locality data (FMNH 40032), Cuzco Amazonico, 15 km E Puerto Maldonado (FMNH 168378, KU 204922, 207785, 220194), Puerto Maldonado (AMNH 56140, 56141, LSUMZ 35249), ca. 30 km SSW of Puerto Maldonado, Tambopata Reserve, Explorer's Inn (USNM 222357, 247511, 247512, 269032). DEPARTAMENTO DE SAN MARTIN: Moyobamba (AMNH 52961), Pachisa (AMNH 52564, 52567). UCAYALI: Pucalpa (=Pucallpa) (IBSP 21833, 21834), Pucallpa, Requena, Monte Carmelo (AMNH 55512, 55515, 55574, 55604, 55612, 55621, 55623, 55644, 55637, 56036), Roaboya (AMNH 52233, 52559, 52892, 53088, 53443, 54421, 54430, 54436, 54443, 54444), Roaboya, Río Ucayali (AMNH 52231, 52544, 55697, 56115), Yarinacocha, Pueblo San Francisco (USNM 238382), Yarinacocha, Ucayali River (FMNH 45573).

***Leptophis occidentalis* (n=231).** COLOMBIA: ANTIOQUIA: 10 km ESE Bolívar, San Juan River Valley, 60 km SW Medellín (AMNH 119799), S of Turbo near the Currulao River (AMNH 94342), Medellín (AMNH 35703, 35704, 35715, 37514, 37936). BOLÍVAR: Gambote, road Cartagena-Sincelejo (FMNH 165501), Gulf of Morrosquillo, west of Sincelejo, Village at sea shore (FMNH 165486). BOYACÁ: Muzo (FMNH 28327). CALDAS: Pueblo Rico, Saint Cecilia (FMNH 54971–54973). CHOCÓ: Andagoya, Condoto area (UMMZ 121053), Jurado (USNM 151637, 151673). CUNDINAMARCA: Apulo (UMMZ 78275). MAGDALENA: banana belt, Rio Frio, Ciénaga (MCZ 25046), Aracataca (ANSP 19784), Bonda (CM 141), Cacagualito (a farm) (CM 2011). META: Villavicencio (MZUSP 5996, 6032, USNM 195928). VALLE DEL CAUCA: Calima River, 7 km from lumber Camp (FMNH 165452*, 165565). COSTA RICA: ALAJUELA: Cariblanco (KU 34043), San Carlos (UMMZ 131431). CARTAGO: mountain above Turrialba, about 6 km NE (CM 69116), Turrialba (KU 30996, 34044, 35642, 34898), Turrialba, Institute of Agiculture (AMNH 69712), Turrialba, IICA (=Inter-American Institute for Agricultural Sciences) (MCZ 56090), 2.5 km E and 4 km N Chitaria (MVZ 79623**), 1.4 mi SW Chitaria via Hwy 233 (MVZ 80023), 2 mi SE of Turrialba (USNM 129072). GUANACASTE: Pozo Azul (AMNH 17363), Tenorio (KU 34902), Tilarán (KU 35643), 2 km E Tilaron (KU 86582). HEREDIA: Río La Selva Biological Station (MVZ 215639), La Selva Field Station, E of Puerto Viejo on Puerto Viejo River (UMMZ 145883), 10 km WSW Puerto Viejo de Sarapiqui (MVZ 217614*). LIMON: Barra del Colorad Island (AMNH 12670, KU 100627), Comadre, off Costa Rica Route 32 (CM 107359), La Lola (UMMZ 117686*), Portete, ca. 5 mi N Limon (AMNH 90625), Santa Marta de Siquirres (ANSP 32396), vicinity of Siquirres (ANSP 32397*). PUNTARENAS: Buenos Aires de Terraba (=Buenos Aires) (FMNH 2527), Golfito (KU 31958, 63841, MCZ 69183), Gremaco, 15 km ESS Potrero Grande (LSUMZ 8758), Julieta, La Ligia Farm (LSUMZ 9635), Monteverde (FMNH 229917), S of San Vito, Estación Biológica Las Cruces (UMMZ 128818), 20 km N of Siquirres, Hamburgo Farm, Santa Clara, between Reventazon and Parismina Rivers (USNM 68859), 4 km N, 2 km W Dominical (KU 63840), 2 km of Rincon de Osa, Osa Tropical Science Center, collected 100 m N of Casa de Santos (USNM 219593), 1.9 mi S Tarcoles River, Hwy 34, north branch of Quebrada Bonita (TCWC 83392), 1.9 mi S Río Tarcoles, Barranca (FMNH 35893). SAN JOSÉ: near Parque Nacional Carara, Bajo Carara on road from Bajo Carara to Sur (TCWC 84615), Quepos (KU 125480). ECUADOR: no locality data (UTA 23523), Maputo Farm (UMMZ 88913), W Ecuador (BMNH 1946.1.6.62). GUAYAS: Durán (USNM 211034), Santiago de Guayaquil (USNM 12269, 12272), 3 mi E of Milagro, San Miguel Farm (USNM 211035). EL ORO: Rosa Delia (USNM 60524), ca. 20 km (airline) E Pasaje, on Machala-Cuenca Road (AMNH 119837), 10 km SE Machala (AMNH 113022). NAPO: Jatuncocha (a lagoon) (USNM 287931). HONDURAS: no locality data: (AMNH 47013**, 47014**). ATLÁNTIDA: on mainland opposite Hog Island (UTA 15868, 53196). GRACIAS A DÍOS: Crique Ibantara (USNM 563500), Kaska Tingni (=Río Kaska) (USNM 559661–559664*), Brus Laguna, Mocorón (UTA 53173, 53511), Palacios (BMNH 1985.1301), Coco River (formely known as the Segovia River) (USNM 24531, 24532), Rus Rus River (USNM 559666, 561111). OLANCHO: confluence between Wampu and Aner Rivers (USNM 319958), 1.5 km N Catacanas (LSUMZ 28447*). NICARAGUA: Kukra Hill (AMNH 12667, 12668), Cupitna Camp (a lumber base on one of the effluents of the Prinzapolka) (AMNH 12718, 12669). MATAGALPA: Matagalpa, La Cumplida Farm (UMMZ 117685). RÍO SAN JUAN: Machuca (ANSP 5288), San Juan de Nicaragua (USNM 15626, 19569, 19570). San Miguelito (AMNH 12666). ZELAYA: Wounta Haulover, Mosquito Coast (ANSP 15434), Siquia River, 7 mi above Rama (UMMZ 79768, 149837***). REGIÓN AUTÓNOMA DEL ATLÁNTICO: Musawas, Waspuc River (AMNH 75224–75427) Topaz mine, 90 mi NW of Bluefields (USNM 37344). PANAMÁ: Galeta Road (MCZ 69475), 14.4 km SSW Bejuco (KU 107775). CANAL DE PANAMÁ: Barro Colorado Island (FMNH 13413, KU 75724, UMMZ 124164, USNM 89401), Empire (Empire Reach) (USNM 59928), Farfan Navy Base (KU 110644), Fort Sherman (KU 107769), Galeta Island (KU 107770), Gamboa (AMNH 32816), Gatun (FMNH 16743, USNM 50106), HWY to Gamboa from Galliard (UMMZ 148006), Juan Mina, Chagres River (KU 107772, 107773), Lower Chilibre River near Panamá Canal Zone BNDY Line (UMMZ 95337), near Fort Kobbe (USNM 140700), Summit (FMNH 131313, 130735, UMMZ 155694, 155718), Tabernilla (a former town that was covered by Lake Gatun) (FMNH 16744), 2.4 mi NNW Summit Gardens (UMMZ 152914). BOCAS DEL TORO: Almirante (KU 80227, 80228, 80229, 80230, 107766), Colón Island, La Gruta (KU 107768, USNM 338257), Punta de Pena (Punta Peña), almost 7 mi inland from village of Chiriqui Grande (USNM 38671), Punta de Pena (Punta Peña), Changuinola River near Quebrada El Guabo, 16 km airline W Almirante (AMNH 119080*), Sibube (USNM 150025), vicinity of Almirante (MVZ 149581*, MVZ 149582, USNM 279079), 1.5 mi W Almirante (KU 107767), 11 km NW Almirante (FMNH 154032). CHIRIQUI: Obispo Station, on railway (MCZ 2719), Progreso (UMMZ 57907, 149836**, 161239), vicinity N of Boquete (CAS 78911–78921, 79034, 79035). COCLÉ: Aguadulce (AMNH 67060), El Valle de Antón (CAS 98550, FMNH 42560, FMNH 47461, MVZ 66606). COLÓN: no locality data (USNM 38506), Achiote (KU 75726), Ciricito (CAS 71425), ca. Coco Solo, east side of Madden Lake (USNM 193392), Culebra (UMMZ 61285), Margarita (UMMZ 152915). DARIÉN: Atlantic side of Isthmus of Panamá (USNM 24504), El Real de Santa Maria (KU 107776), Jaqué (USNM 161229), Cerro Tacarcuna (KU 75727, 75728*), Yaviza (MVZ 83434, 83435). PANAMÁ: Ancón (USNM 51909), ca. around Panamá city (FMNH 216246), Cerro Azul región-Cerro Jéfe región, Piedras-Pacora Ridge (AMNH 119881), Chepo (USNM 48958), Chepo, La Joya, pacific side (ANSP 25078), Las Cumbres (KU 110643), Nueva Gorgona (AMNH 90038–90051), S slope Cerro La Campana (KU 75725*), San Blas, Camp Sasardi (KU 110642), Venado Beach (USNM 193377, 193378, 531871, 531872), 5 km NW Pacora (KU 107774). PANAMÁ OESTE: La Chorrera (FMNH 16742). LOS SANTOS: no locality data: (USNM 51910), La Jagua (USNM 129927), Las Palmitas (USNM 148220), Panamá city (USNM 50124). VERAGUAS: Mojarra (USNM 129874, 129875). GUNA YALA: Armila (USNM 150132, 150133). PERU: CAJAMARCA: Zana River, Monte Seco-Udima Road, 1 km E El Chorro (ANSP 31790). PIURA: a 1.5 km (road) E Canchaque on Huancabamba Road (LSUMZ 32565). VENEZUELA: VARGAS: Macuto (USNM 27821, paratype of *L*. *coeruleodorsus*, reidentified as *L*. *occidentalis* in the present study). AMAZONAS: San Juan de Manapiare, Cerro Morrocoy (MHNLS 1631). SUCRE: 3–5 km N Macuro (KU 133535). TÁCHIRA: Caraperro River, San Félix) (UMMZ 55895), San Cristóbal (MHNLS 1086). YARACUY: Aroa (UMMZ 55896).

***Leptophis praestans* (n=90).** BELIZE: BELICE: Belice (AMNH 126430). CAYO: Central Farm (MCZ 71648), Cohune Ridge (UMMZ 80713), Coquericot (UMMZ 74905). STANN CREEK: 4 mi W Dangriga (CM 112117). TOLEDO: about 2 mi SE Big Falls, Grande River (CM 105883), Bladen Nature Reserve, Ek Xux Camp on Bladen Branch (USNM 498219), Bladen Nature Reserve, Richardson Creek Camp on Richardson Creek (USNM 498218), Bladen Nature Reserve, Teakettle Camp on Bladen Branch (USNM 496721, 496722), Blue Creek Village, vicinity of Slattery Field Station (UTA 11068*, 11069*, 12685). GUATEMALA: no locality data: (USNM 6754a, 6754b). ALTA VERAPAZ: Chamá Farm (UMMZ 89974), San Juan Farm (UTA 46627). IZABAL: Bobos plantation, near Playitas (FMNH 20089), Las Escobas (a waterfall), 5.1 km W Santo Tomás (de Castilla) (KU 187326*, 191000, 191002, 191003, 190999), Los Amates, Aldea Vista Hermosa (KU 190991), Los Amates, Aldea Vista Hermosa (KU 190992, 190994, 190995, 190996, 190997, 190998), Los Amates, Sierra del Espírito Santu, Aldea Santo Antonio (UTA 28406), Montañas del Mico, Las Escobas, 5.1 rd km WSW Puerto Santo Tomás (de Castilla) (UTA 28405, 28634), Morales, Sierra Caral, Aldea San Miguelito (UTA 38124), Morales, Sierra Caral, Carreteria Quebradas-San Miguelito, Acampamento 1.160 m (UTA 37057), Morales, Sierra Caral, road between Quebradas-San Miguelito, Primer Acampamento (UTA 37056), Quebradas (FMNH 20489), Sierra de Santa Cruz, Semuc Farm, headquarters, near Semuc River (UTA 26217), Sierra de Santa Cruz, Semuc Farm, ca. 1 km S headquarters, near Semuc River (UTA 22175*, 26659), Sierra de Santa Cruz, Semuc River, La “35” (UTA 28407, 28408). PETÉN: La Libertad (UMMZ 74904, 79053, 149839**), La Libertad, Parque Nacional Sierra del Lacandón, Yaxchilán (UTA 46134**), La Libertad, 1 mi N Subín River, Saint Tereza (UMMZ 74851), 13 km NNW Chinajá (KU 55716*, 57161), Nueva Agua (USNM 71366), Tikal (UMMZ 124341, 124342, UTA 35018), ca. 10 km SW of main aguada at Tikal on El Cruce-Tikal Road (UTA 39227). ZACAPA: Gualán, Aldea Dona Maria (KU 190993). HONDURAS: through NYZS, no locality data: (AMNH 32341). ATLÁNTIDA: along Río Cangrejal, ca 12 km SSE La Ceiba (KU 101449*), Lancetilla (located in the municipality of Tela) (AMNH 46973*, 46974), Tela (USNM 84259), Tela, Lancetilla (USNM 120831), Parque Nacional Pico Bonito, Oro stream, tributary of Viejo River (USNM 559667), 6.5 mi E La Ceiba, mts above Piedra Pintada (LSUMZ 28401). COLÓN: 5 km SW Trujilo (LSUMZ 21754). CORTÉS: W of San Pedro, Santa Ana, bottom of Santa Ana Canyon (FMNH 5292**, 5293, 5294**), 3 mi W of San Pedro Sula, Santa Ana Canyon (LSUMZ 28400). YORO: El Progreso (MCZ 26859*). MEXICO: CAMPECHE: near Xpujil (KU 301067). CHIAPAS: Florida, 50 km E Altamirano (TCWC 19544), Palenque ruins (KU 94133), Solosuchiapa 3.8 mis S (UMMZ 126202), Solosuchiapa, 4.6 mis N (UMMZ 126203). OAXACA: Metates, Sierra de Juárez (UTA 12358, 14212). QUINTANA ROO: Xkanha, Campeche-boundary (AMNH 7861), Felipe Carrillo Puerto (UMMZ 113571), 0.7 mi S Tulum Pueblo (LSUMZ 38599), 35 km NE Felipe Carrillo Puerto (KU 301068). TABASCO: Macuspana (KU 41603), 31 mi W of Cardenas (AMNH 88836), 7.2 mi W Cardenas (LSUMZ 33367). VERACRUZ DE IGNACIO DE LA LLAVE: Cuezolapan, Arroyo Ahuacapan (TCWC 21980), Lower falls of Quetzalapan River (TCWC 19155), San Andrés Tuxtla, Dos Amates (CM 52758), Veracruz (FMNH 208220), 7.7 mi NW Sontecomapan by road (UTA 2643*), 16.4 mi N Juan Diaz Covarrubias (UTA 2775). YUCATÁN: Santa Maria Colony (located in the municipality of Mérida) (UMMZ 76165).

***Leptophis urostictus* (n=15).** COLOMBIA: CAUCA (one of the departments of Gran Colombia, 1819-1831): Quebrada Guanguí, 0.5 km above Patía River (upper Saija drainage) (AMNH 107930, 107935, 109751, 109752, 109753*, 109754). CHOCÓ: Condoto, Peña Lisa (AMNH 8062, BMNH 1914.5.21.38, MCZ 13298), Pizarro (FMNH 43737), Condoto River, Camp Peñalisa, eastern tributary of San Juan River (UMMZ 55528**), San Juan River (USNM 159496). RISARALDA: about 7 km SE Santa Cecilia, south bank San Juan River (AMNH 115932, 115933). VALLE DEL CAUCA: Llanobajo (KU 169951).
