# Supplementary material for: A new species of parrot snake, Leptophis (Serpentes: Colubridae) from the Brazilian Cerrado
Source: PeerJ. 2025 Jan 30;13:e18528. doi: 10.7717/peerj.18528 (PMC11787803; doi:10.7717/peerj.18528)
Supplement: Supplemental Information 3 [file peerj-13-18528-s003.docx]

**Supplementary Material 2.** GenBank accession numbers for sequence data, specimen voucher numbers, collecting locality information, and references for all samples included in molecular analyses.

| **Species** | **Genbank** | **Voucher Number** | **Country** | **State/Province/Locality** | **Reference** |
| --- | --- | --- | --- | --- | --- |
| *L. ahaetulla* | KF667671 | AMNH140899 | Guyana | Dubulay Ranch on the Berbice Rive | Murphy *et al*. (2013) |
| *L. ahaetulla* | PP463857 | MAP177 | Brazil | Amazonas, Parintins | Present Work |
| *L. bocourti* | MZ126814 | QCAZ7362 | Ecuador | Santo Domingo de los Tsachilas | Torres-Carvajal *et al*. (2021) |
| *L. bocourti* | MZ126815 | QCAZ8023 | Ecuador | Esmeraldas: Durango. 1.042 -78.624 | Torres-Carvajal *et al*. (2021) |
| *L. bocourti* | MZ126816 | QCAZ16942 | Ecuador | Esmeraldas: Mache-Chindul Ecological Reserve, Laguna de Cube | Torres-Carvajal *et al*. (2021) |
| *L. coeruleodorsus* | KF667666 | CAS245120 | Trinidad and Tobago | Tobago, near the Bloody Bay river | Murphy *et al*. (2013) |
| *L. coeruleodorsus* | KF667672 | UWIZM2012.85 | Trinidad | Arima Valley | Murphy *et al*. (2013) |
| *L. coeruleodorsus* | KF667673 | CAS231717 | Trinidad | Trinidad, junction of road ER16 with San Fernando Road | Murphy *et al*. (2013) |
| *L. coeruleodorsus* | KF667674 | CAS231809 | Trinidad | Trinidad, tributary of River Cuesa | Murphy *et al*. (2013) |
| *L. cupreus* | MZ126823 | QCAZ15912 | Ecuador | Zamora Chinchipe: Mirador ECSA mining concession | Torres-Carvajal *et al*. (2021) |
| *L. cupreus* | MZ126824 | QCAZ16082 | Ecuador | Morona Santiago: Kunkuk Shuar community | Torres-Carvajal *et al*. (2021) |
| *L. depressirostris* | KR814643 | FN253772 | No data available | No data available | Pyton, R.A. Not published |
| *L. depressirostris* | KX660270 | LSUMNS146385 | No data available | No data available | Figueroa *et al*. (2016) |
| *L. depressirostris* | MH140830 | CH5351 | Panama | Colon, Quebrada Cedro Hueco | Mulcahy *et al*. (2022) |
| *L. depressirostris* | MH140831 | USNMFS195526 | Panama | Bocas Del Toro, Isla Escudo de Veraguas | Mulcahy *et al*. (2022) |
| *L. depressirostris* | MH140832 | CH8812 | Panama | Colon, Donoso, Cocle del Norte | Mulcahy *et al*. (2022) |
| *L. depressirostris* | MZ126825 | QCAZ6670 | Ecuador | Esmeraldas: Alto Tambo, El Placer, Carolina river. | Torres-Carvajal *et al*. (2021) |
| *L. depressirostris* | MZ126826 | QCAZ8792 | Ecuador | Esmeraldas: Alto Tambo | Torres-Carvajal *et al*. (2021) |
| *L. depressirostris* | MZ126827 | QCAZ14497 | Ecuador | Imbabura: Cachaco-Lita | Torres-Carvajal *et al*. (2021) |
| *L. depressirostris* | MZ126828 | QCAZ16941 | Ecuador | Manabí: Mache-Chindul Ecological Reserve | Torres-Carvajal *et al*. (2021) |
| *L. dibernardoi* | ON123616 | URCA12125 | Brazil | Mauriti, CE | Albuquerque *et al*. (2022) |
| *L. dibernardoi* | ON123618 | URCA10126 | Brazil | Ceará, Crato municipality | Albuquerque *et al*. (2022) |
| *L. diplotropis* | KX660271 | LSUMZ6328 | Mexico | Colima | Figueroa *et al*. (2016) |
| *L. marginatus* | KF667675 | UAM:H-103 | Paraguay | Lagunitas, Mbaracayu Reserve | Murphy *et al*. (2013) |
| *L. marginatus* | MN276246 | GK3594 | Paraguay | Concepcion | Cacciali *et al*. (2019) |
| *L. marginatus* | MN276247 | GK3809 | Paraguay | Concepcion | Cacciali *et al*. (2019) |
| *L. marginatus* | MN276248 | PCS532 | Paraguay | Guaira | Cacciali *et al*. (2019) |
| *L. marginatus* | PP463856 | MAP1830 | Brazil | Mato Grosso do Sul, Aquidauana | Present Work |
| *L.* *mystacinus* sp. nov. | HM582222 | No data available | No data available | No data available | Klaczko *et al*. (2014) |
| *L.* *mystacinus* sp. nov. | MK209302 | MZUSP18646 | Brazil | Tocantins, Palmas | Montingelli *et al*. (2019) |
| *L.* *mystacinus* sp. nov. | PP463855 | MAP3430 | Brazil | Caseara, Tocantins | Present Work |
| *L.* *mystacinus* sp. nov. | PP463854 | MAP3211 | Brazil | Caseara, Tocantins | Present Work |
| *L. nigromarginatus* | KF667667 | LSUMZ14017 | Brazil | Amazonas state, Rio Ituxi at the Madeirera Scheffer | Murphy *et al*. (2013) |
| *L. nigromarginatus* | KF667670 | LSUMZ12288 | Brazil | Amazonas state, 1 km east of Waimari Indian Reserve | Murphy *et al*. (2013) |
| *L. nigromarginatus* | MZ126817 | QCAZ4247 | Ecuador | Sucumbíos: Bermejo river | Torres-Carvajal *et al*. (2021) |
| *L. nigromarginatus* | MZ126818 | QCAZ14743 | Ecuador | Orellana: Yasuni National Park | Torres-Carvajal *et al*. (2021) |
| *L. nigromarginatus* | MZ126819 | QCAZ16670 | Ecuador | Esmeraldas: Alto Tambo, El Placer, Carolina | Torres-Carvajal *et al*. (2021) |
| *L. nigromarginatus* | MZ126820 | QCAZ17489 | Ecuador | Orellana: Pozo Amo | Torres-Carvajal *et al*. (2021) |
| *L. nigromarginatus* | KF667669 | LSUMZ13874 | Brazil | Acre state, ~ 5 km north of Poto Walter | Murphy *et al*. (2013) |
| *L. occidentalis* | MH140827 | CH8811 | Panama | Colon, Donoso, Cocle del Norte | Mulcahy *et al*. (2022) |
| *L. occidentalis* | MH140828 | JM776 | Panama | Cocle, El Cope | Mulcahy *et al*. (2022) |
| *L. occidentalis* | MH140829 | USNMFS195481 | Panama | Bocas Del Toro, Isla Escudo de Veraguas | Mulcahy *et al*. (2022) |
| *L. occidentalis* | MZ126821 | QCAZ6393 | Ecuador | El Oro: Arenillas, Huaquillas, La Cuca | Torres-Carvajal *et al*. (2021) |
| *L. occidentalis* | MZ126822 | QCAZ7443 | Ecuador | Loja: Alamor-Arenillas road | Torres-Carvajal *et al*. (2021) |
| *L. occidentalis* | KF667668 | LSUMZ6359 | Honduras | No specific locality given | Murphy *et al*. (2013) |
| *L. riveti* | MZ126829 | QCAZ12439 | Ecuador | Azuay | Torres-Carvajal *et al*. (2021) |
| *L. riveti* | MZ126830 | QCAZ15107 | Ecuador | El Oro: Chilla | Torres-Carvajal *et al*. (2021) |
| *L. riveti* | MZ126831 | QCAZ16943 | Ecuador | Esmeraldas: Mache-Chindul Ecological Reserve | Torres-Carvajal *et al*. (2021) |
| *Chironius scurrulus* | KX660266 | LSUMNS9368 | Bolivia | La Paz | Figueroa *et al*. (2016) |
| *Dendrophidion dendrophis* | MK086632 | QCAZ13824 | Ecuador | Zamora Chinchipe | Torres-Carvajal *et al*. (2019) |
| *Lampropeltis californiae* | KU323980 | No data available | No data available | No data available | Simões *et al*. (2016) |
