# Supplementary figures and images for: A new species of parrot snake, Leptophis (Serpentes: Colubridae) from the Brazilian Cerrado
Source: PeerJ. 2025 Jan 30;13:e18528. doi: 10.7717/peerj.18528 (PMC11787803; doi:10.7717/peerj.18528)

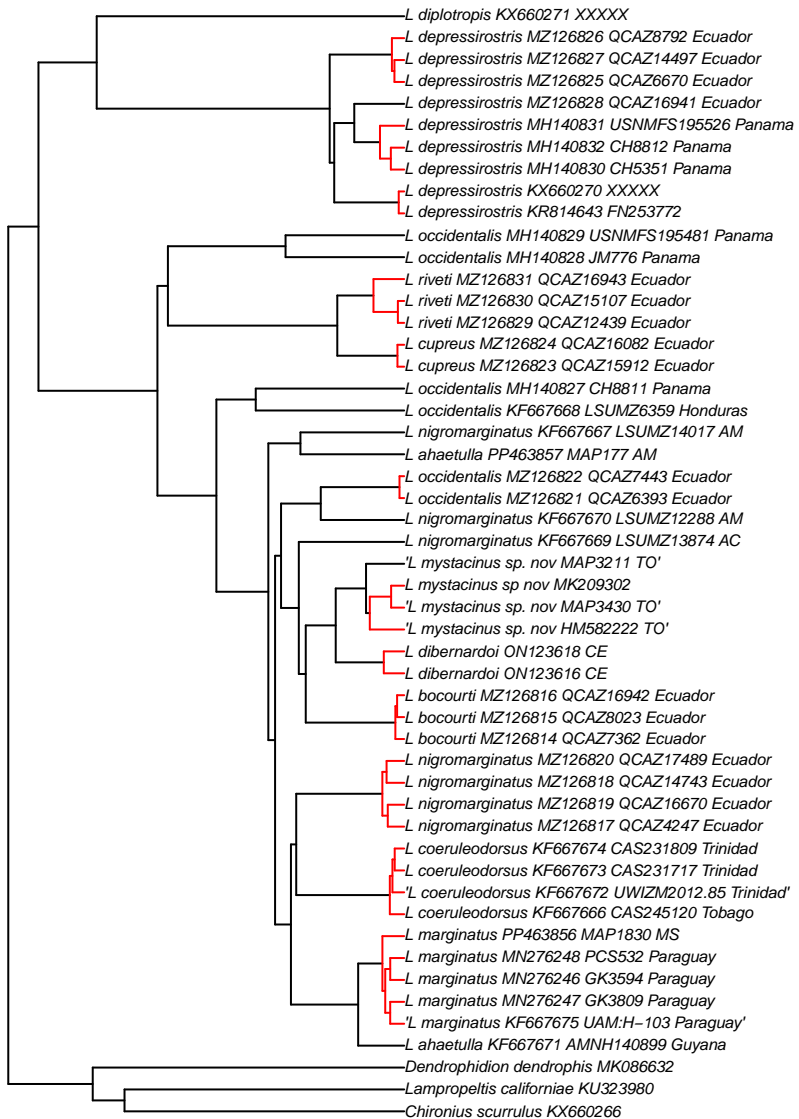

Supplement: Supplemental Information 4 — Tree with the GMYC results. Branches connected in red belong to the same evolutionary entity. [file peerj-13-18528-s004.pdf]
